# Programmed cell death mechanisms of traditional plant medicine in prostate cancer therapy

**DOI:** 10.3389/fimmu.2026.1833601

**Published:** 2026-07-14

**Authors:** Yuchen Zhang, Sheng Qin, Yingping Wang, Yajuan Xu, Yanzhu Zhu

**Affiliations:** 1 Key Laboratory of Medicinal Materials, Jilin Academy of Chinese Medicine Sciences, Changchun, China; 2 College of Traditional Chinese Medicine, Jilin Agricultural University, Changchun, China; 3 Jilin Agricultural University, State Local Joint Engineering Research Center of Ginseng Breeding and Application, Changchun, China; 4 Key Lab of Preventive Veterinary Medicine in Jilin Province, College of Animal Science and Technology, Jilin Agricultural Science and Technology University, Jilin, China

**Keywords:** anti-cancer activity, apoptosis, programmed cell death, prostate cancer, traditional plant medicine

## Abstract

**Background:**

Prostate cancer (PCa) is a prevalent malignancy in males with high morbidity and mortality. Although treatment modalities have evolved considerably, tumor resistance, recurrence, and metastasis persist, urgently requiring the exploration of alternative therapies for PCa. There is ongoing research on finding and identifying the use of traditional plant medicine (TPM). Cellular homeostasis comprises a sophisticated network of metabolic processes that functions cooperatively to preserve a stable intracellular environment. Programmed cell death (PCD) plays an important role in PCa mechanism. Thus, they represent an effective strategy for targeting PCa. TPM has been proven to induce PCD through multiple pathways and target in the treatment of PCa. Recent reviews have only focused on the one of the PCD, and autophagy, apoptosis, pyroptosis, ferroptosis, and necroptosis are not simultaneously reviewed.

**Methods:**

The search strategy: articles with the title containing “prostate cancer”, “therapeutic”, “traditional medicine”, “apoptosis”, “pyroptosis”, autophagy”, “*in vivo/in vitro*”, “active ingredients”, “Herbal”, “real modules”, “dose”, “pathway”, “effects/mechanisms”, “extract”, “pure compound”, “drug type”, “anticancer activity”, “Chinese herbal compounds”, “necroptosis” and “ferroptosis” had been initially selected in the past five years databases of PubMed, Web of Science, and ScienceDirect. The references were screened according to the strategy.

**Results:**

Forty-two drugs in the TPM have been chosen in this review. The plant extract, Chinese herbal compound, and pure compound of TPM exhibit significant anticancer activity against PCa by regulating multiple kinds of PCD. More importantly, PI3K/AKT/mTOR, AMPK/mTOR pathways, AKT1/Bcl2/NF-κB, GPBAR1/NF-κB, Keap1/Nrf2/ARE, PINK1/Parkin signaling pathways serve as critical molecular targets mediating the anticancer activities of TPMs in PCD. Autophagy, apoptosis, and ferroptosis are research hotspots, while pyroptosis and necroptosis are less explored. Apoptosis is co-detected with autophagy, or necroptosis. Ferroptosis is co-detected with necroptosis, or pyroptosis. Notably, the interrelationships between these cell death modes are rarely investigated in depth in the treatment of TPM in PCa. TPM has been induced apoptosis, ferroptosis, necroptosis in PCa. But the effect of TPM on the autophagy and pyroptosis need further evidence to clarify the mechanism.

**Conclusion:**

Hence, it is imperative to focus on elucidating the role of PCD modulators to refine therapeutic strategies of TPM in PCa.

## Introduction

Prostate cancer (PCa), a prevalent malignancy among men worldwide, significantly contributes to increased mortality rates in men globally, and poses a serious threat to men’s lives and health (1). Over the past five years, PCa mortality has risen across Latin America, Asia, Africa, Eastern Europe and the Caribbean (2). In 2022, PCa has accounted for an estimated 1, 414, 259 incident cases and 375, 304 cancer-related deaths worldwide (3). In men worldwide, an estimated 288, 300 new cases of PCa were diagnosed in 2023 (4). Totally, 10%–20% of metastatic patients develop castration-resistant PCa (CRPC) within five years (5), increasing the mortality burden in this population. The pathogenesis of PCa include tumor cell proliferation, metastasis, programmed cell death (PCD), and chemotherapy resistance (6). In PCa treatments, radiation therapy, active surveillance, hormonal therapy, surgery, chemotherapy, immunotherapy and cryotherapy are the main methods (7). Current PCa therapies demonstrate limited clinical benefit, with routine practice hampered by prevalent therapeutic resistance and substantial treatment-related toxicities. Therefore, new alternative drugs are being explored as supportive therapeutic agents.

Traditional plant medicines (TPM) have demonstrated efficacy in suppressing tumor cell proliferation and metastasis, inducing apoptosis, modulating autophagy and ferroptosis, and overcoming chemotherapeutic resistance (8). TPM has confirmed the anti-cancer effectiveness in treating PCa (9). Medicinal formulas, extracts, and pure compounds of TPM have been found to inhibit the development of PCa (10). Recent studies have identified PCD as new targets for PCa treatment. PCD serves as a critical surveillance mechanism for eliminating mutated cells with neoplastic potential, thereby preventing malignant transformation and tumorigenesis. It is helpful to overcome the therapeutic resistance of PCa. PCD includes apoptosis, pyroptosis, autophagy, ferroptosis and necroptosis, which are effective strategies for targeting PCa. TPM exerts anticancer effects on PCa by regulating multiple forms of PCD, including autophagy, apoptosis, pyroptosis, ferroptosis, necroptosis, entosis, cuproptosis, disulfidptosis, and PANoptosis (11–13). Entosis, cuproptosis, disulfidptosis, PANoptosis are recently characterized form of PCD and regulates microenvironment in PCa. However, limited data showed the effect of TPM on the entosis, cuproptosis, disulfidptosis, and PANoptosis in PCa. No reviews have been conducted to explore the induction of PCD of TPM in PCa and explore the relationship between them.

Thus, the mechanisms of apoptosis, pyroptosis, autophagy, ferroptosis, and necroptosis are delineated in TPM treatment of PCa. The TPM includes plant extract, Chinese herbal compound and pure compound. This review will offer a promising adjunctive approach and treatment strategy for PCa and clarify the underlying mechanisms. Thus, therapeutic strategies of TPM aimed at PCD represent promising avenues to circumvent treatment resistance and enhance therapeutic efficacy in PCa.

## Methods

### The search strategy

**Figure 1:**
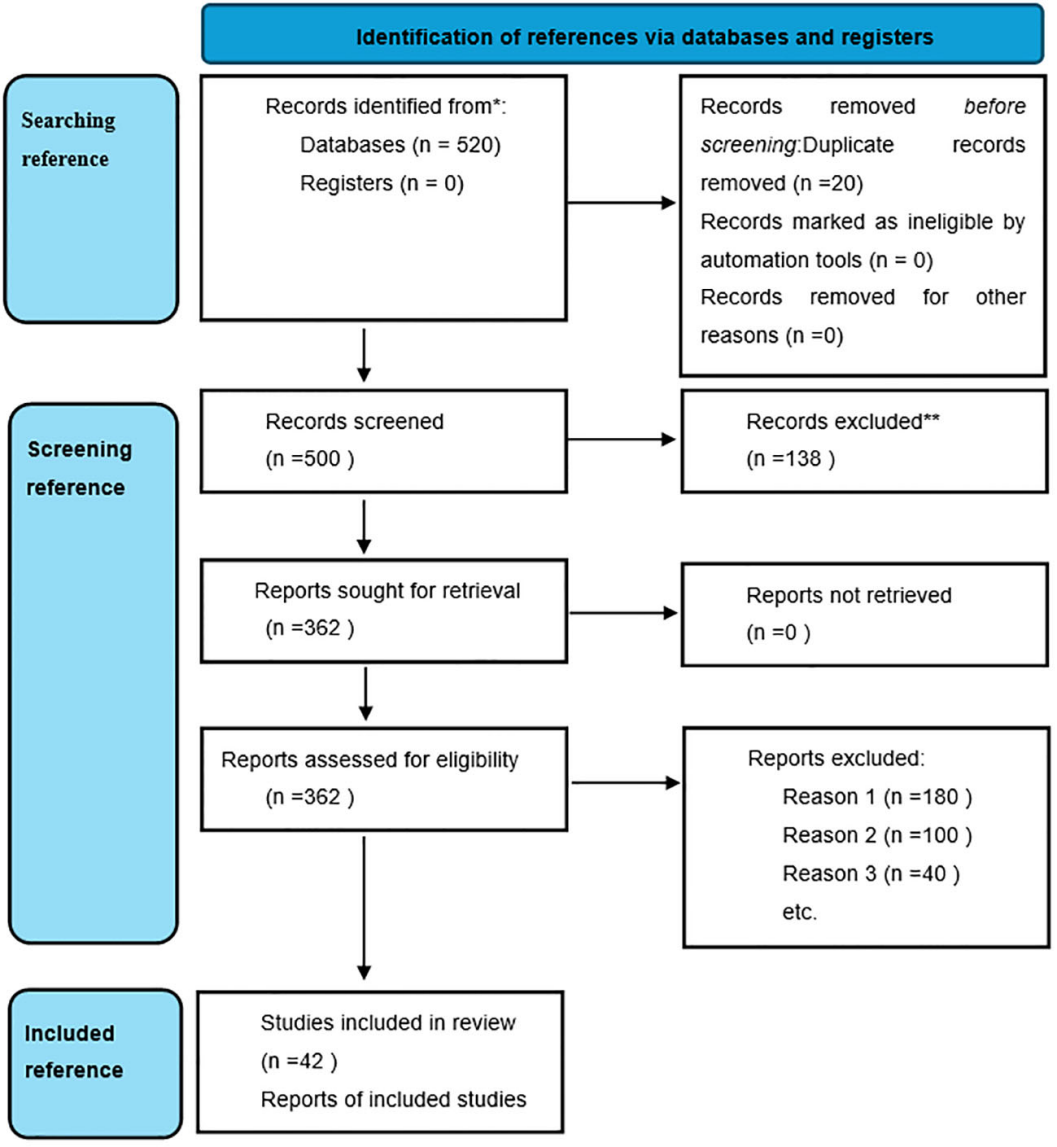
The screening strategy of the references. The step of searching references is conducted according to the search strategy. The screening references is conducted according to the inclusion and exclusion criteria. The included references are identified by the data extraction and quality assessment.

articles with the title containing “prostate cancer”, “therapeutic”, “traditional medicine”, “apoptosis”, “pyroptosis”, autophagy”, “*in vivo/in vitro*”, “active ingredients”, “Herbal”, “real modules”, “dose”, “pathway”, “effects/mechanisms”, “extract”, “pure compound”, “drug type”, “anticancer activity”, “Chinese herbal compounds”, “necroptosis” and “ferroptosis” had been initially selected in the past five years databases of PubMed, Web of Science, and ScienceDirect. The references were screened according to the strategy (**Figure 1**). The figures in this experiment were created with BioGDP.com and are authorized to use in this manuscript (14).

### Inclusion criteria

Publication within the past five years, focus on the anti-cancer mechanisms of TPM in PCa, involvement of at least one form of PCD, investigation of plant extracts, Chinese herbal compounds, or pure compounds, and publication in peer−reviewed journals with complete experimental data.

### Exclusion criteria

Reviews, meta-analyses, letters, conference abstracts, and case reports were excluded; studies irrelevant to PCa or TPM–mediated PCD, duplicate, low-quality, or data-incomplete publications, and non-English articles with inaccessible full text were excluded.

### Data extraction

The basic study information, types and sources of TPM, experimental models, regulated cell death types, signaling pathways, molecular markers, and core conclusions were collected.

### Quality assessment

Quality assessment was performed by evaluating the rationality of experimental design, standardization of detection methods, reliability of mechanistic verification, completeness of data, and journal authority to ensure the validity of included studies.

## The analysis of the included references

**Figure 2:**
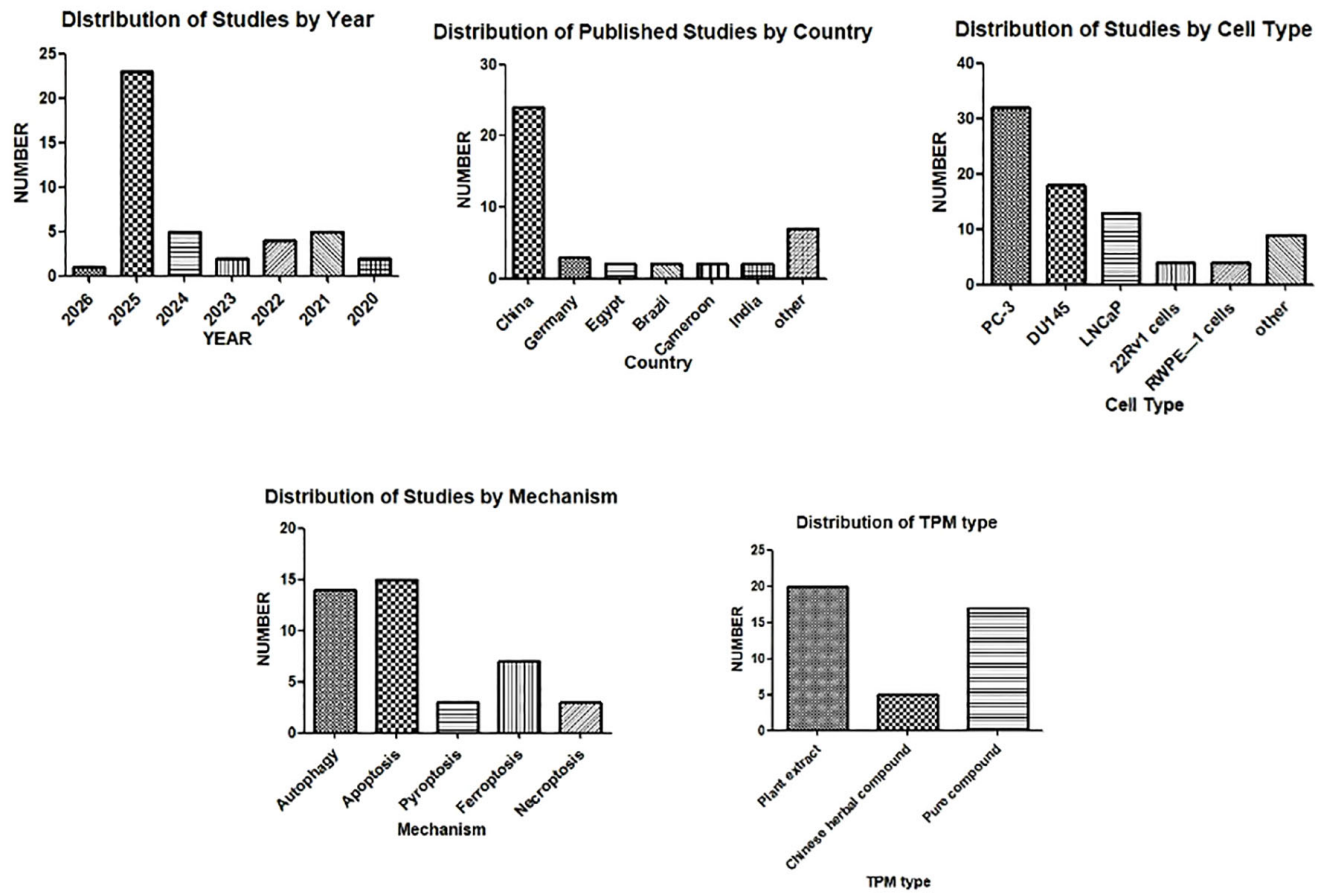
The analysis of the included references. Forty-two references are calculated by the year, country, cell type, mechanism and TPM types.

A total of 42 valid studies on PCa were included in the statistical analysis, covering the period from 2020 to 2026 (**Figure 2**). Temporally, the distribution of studies showed a clear concentration trend: 2025 had the highest number of publications (23 studies, 54.8%), followed by 2021 and 2024 (5 studies each, 11.9%), 2022 (4 studies, 9.5%), 2020 and 2023 (2 studies, 4.8%), and 2026 (1 study, 2.4%), indicating a surging research focus in recent years, especially in 2025. Geographically, China contributed the most studies (24 studies, 57.1%), which was significantly higher than other countries; Germany contributed 3 studies (7.1%), while Egypt, Brazil, Cameroon, and India each contributed 2 studies (4.8%); other countries (Greece, Türkiye, Tanzania, Tunisia, South Korea, Iran, and Turkey) had 1 study (2.4%), respectively. In terms of cell models, PC-3 cells were the most commonly used (32 studies, 76.2%), followed by DU145 cells (18 studies, 42.9%), LNCap cells (13 studies, 31.0%), 22Rv1 cells and RWPE-1 cells (4 studies, 9.5%), PC-3-AbiR cells (3 studies, 7.1%), and other cell types (including unspecified prostate cancer cells, CRPC cells, C42 cells, mTOR pathway cells, and xenografted mice cells) were used in 1 study (2.4%), reflecting the widespread preference for PC-3 cells as the classic *in vitro* model in PCa research. Regarding the underlying anti-tumor mechanisms, apoptosis was the most frequently reported regulatory pathway (15 studies, 35.7%), followed by autophagy (14 studies, 33.3%), ferroptosis (7 studies, 16.7%), and pyroptosis and necroptosis (3 studies, 7.1%), respectively, indicating that PCD pathways are the core research focus of natural products against PCa. In terms of the types of tested natural products, plant extracts were the most studied category (20 studies, 47.6%), followed by pure compounds (17 studies, 40.5%), and Chinese herbal compounds (5 studies, 11.9%), showing that plant extracts and pure active compounds are the main research objects in this field.

## Autophagy

Autophagy is an endogenous mechanism that transports cells ‘own cytoplasmic materials and organelles to lysosomes for degradation. LC3B-II belongs to the microtubule-associated protein 1A/1B-light chain 3 (LC3) family and accumulates specifically in newly formed autophagosomes (15). Autophagy represents an evolutionarily conserved lysosomal degradation pathway wherein damaged proteins and cytoplasmic constituents are sequestered and recycled to generate metabolic precursors and maintain energy homeostasis, particularly under nutrient-limiting conditions (16). Autophagy serves pivotal functions in adapting to and combating pathogenic infection, nutrient scarcity, orchestrating antigen presentation, and mitigating oxidative stress (17). Autophagy suppresses proliferation, metastasis, and invasion in various malignancies (18). Today, modulation of autophagy is the anticancer mechanisms of natural products and anticancer agents (19, 20). In PCa, autophagy critically modulates apoptotic signaling and tumor progression via multiple molecular pathways, prominently the PI3K/AKT/mTOR axis (21). Herein, we comprehensively review the current understanding of TPM-mediated autophagy modulation in PCa, aiming to establish a theoretical framework for future investigations.

### Plant extract

Plant extract, the preliminary extraction of TPM, are often used to investigate the pharmacological effects of the plant medicine. Previous studies have implicated plant extract in the antitumor effects of the *Zingiber officinale* (ginger) extract, *Cucurbita pepo* (pumpkin) seed extract, *Thymus vulgaris* (thyme) extract, *Cannabis sativa* (hemp) extract. However, autophagy is not the main topic in their experiment. 6-gingerol at 1-10 µM (equivalent to 0.294–2.944 µg/mL) ginger extract (0.25 mg/mL) has markedly attenuated the expression of autophagy-related proteins LC3B and Atg12, and promote Caspase 3 mRNA expression in PC-3 cells (22). The results indicated that the treatment with ginger extract inhibited autophagy and significantly increased apoptosis. Autophagy inhibition by this mechanism has markedly increased tumor mortality. Pumpkin seed, a rich source of polyphenols and bioactive compounds, demonstrates chemopreventive potential against cancer, likely mediated through autophagy pathway interference. The polar lipid and water extracts of pumpkin seed have significantly induced autophagy in PC-3 cells, indicating that pumpkin seed extracts modulate the autophagic machinery of PC-3 cells (23). The combined treatment with *Thymus vulgaris* extracts and Docetaxel (DTX) has inhibited cell viability, arrested G0/G1 cell cycle, increased apoptosis, and enhanced autophagy by upregulating important autophagy-related markers such as LC3B-II and Beclin1 (24). In PCa, the apoptosis and autophagy are also activated. *Cannabis sativa* extract is one of the most researched extracts, especially among supportive treatments against cancer. Cotreatment of etoposide with cannabidiol (CBD) markedly suppressed autophagic flux and also induced apoptosis through activation caspase-3 and PARP-1 in androgen-dependent PCa cells (4). Ginger extract and CBD have suppressed autophagy, whereas pumpkin seed*, Thymus vulgaris* extract enhanced autophagy. They exert opposite effects in autophagy activation. These four substances represent four distinct phytochemical paradigms: a rhizomatous phenolic ketone, a cannabis-derived terpenophenol, a nutrient-dense seed matrix, and an aromatic monoterpene phenol. Their divergent botanical origins, chemical architectures, and mechanistic targets underscore that their shared influence on autophagy is a functional convergence rather than a reflection of structural or taxonomic relatedness. The present study suggests that the ginger extract, pumpkin seed, *Thymus vulgaris* extract, *cannabis sativa* extract can be used as supportive therapy in existing chemotherapeutic approaches, and they may be a promising option.

Autophagy mechanism is used as the main mechanism to explore in the *calotropis procera* (Giant milkweed) extract (CPE) and *Styrax liquidus* (liquidambar, storax, sweet gum) extract (SLG). CPE treatment has differentially elevated the LC3-II/LC3-I ratio in PC-3 cells, whereas it markedly reduced both LC3-II/LC3-I ratio and beclin-1 expression in 22Rv1 cells relative to vehicle controls (25). There are data suggesting that depending on the cellular features of the PCa cell lines, either induction or inhibition of autophagy can result in cell survival (26). Studies have shown that activation of autophagy in androgen-independent PC-3 cells results in cell cycle arrest in G2/M phase (27, 28). However, it has been observed that autophagy acts as pro-survival mechanism in androgen-responsive 22Rv1 cells. CPE inhibits PCa cell viability possibly by regulating the autophagy pathway and/or regulating ROS levels. However, the mechanism underlying this differential effect of CPE on autophagy remains unclear. SLG extract, enriched with ravidomycin derivatives, potently induced autophagic cell death in PCa cells through PI3K/AKT/mTOR pathway inhibition (29). The signaling pathways involved in autophagy are complicated and include the PI3K/Akt/mTOR pathway that inhibits autophagy (30). Akt is a downstream effector of PI3K that can stimulate the mTOR to regulate autophagy negatively. The PI3K/Akt pathway may be involved in the regulation of autophagy by SLG. However, there were no necrotic cells or fragmented DNA after the extract treatment at 72 h which was the highest cytotoxic time point (10–80 µg/mL). Therefore, SLG extract treatment did not induce apoptosis or necrosis in human PC-3 and DU-145 PCa cells. These results indicated that autophagy was initiated in human PCa cells. Thus, CPE and SLG represent promising alternative therapeutic candidates for PCa management. The mechanism of autophagy includes the autophagy pathway, altering the ROS levels, and PI3K/AKT/mTOR pathway.

### Chinese herbal compound

Chinese herbal compounds, characterized by multifaceted bioactive constituents, have been employed in PCa therapy. Xiaoaiping (XAP) injection, a TPM formulation derived from *Marsdenia tenacissima* (Roxb.), originates from a plant predominantly cultivated in China. This plant is known for its heat-eliminating, diuretic, expectorant, and fire-purging properties. XAP suppresses autophagy by downregulating Atg5/Atg12 conjugation and upregulating FoxO3a expression and its subsequent nuclear translocation (18). In C4–2 and PCa cell line-3 (PC-3) cells, XAP induced cellular apoptosis, evidenced by reduced B-cell lymphoma 2 (Bcl-2) levels and elevated Bcl-2-associated X (Bax) levels. These findings suggest that XAP induces PCa apoptosis via inhibition of FoxO3a autophagic degradation, potentially offering a novel perspective on XAP injection for PCa. When FoxO3a, a non-degradable autophagy substrate, is inhibited in PCa cells, it accumulates and subsequently translocates to the nucleus to activate pro-apoptotic proteins and initiate the apoptosis cascade (31). Qi Ling decoction (QLD), a multi-herb traditional Chinese medicine formulation, QLD restored the abiraterone sensitivity of PC-3-AbiR and DU145-AbiR cells through inhibiting autophagy (32). QLD was capable of promoting abiraterone-induced apoptosis and cell death of PC-3-AbiR and DU145-AbiR cells *in vitro*. While QLD resensitizes abiraterone-resistant PCa cells to therapy, the precise molecular mechanisms underlying its modulation of autophagosome biogenesis remain elusive. Although both XAP and QLD exert anticancer effects through autophagy inhibition in PCa, further mechanistic investigations are warranted to substantiate these findings.

### Pure compound

Pure compounds are purified from TPM extracts. Autophagy is mentioned in the baicalin and celastrol. Baicalin, a natural flavonoid derived from *Scutellaria baicalensis*, exerts antioxidant, anti-inflammatory, and anticancer activities across diverse malignancies, at least in part through autophagy induction (33). Celastrol, a bioactive constituent of *Tripterygium wilfordii* Hook F., exhibits pleiotropic pharmacological properties, including antineoplastic effects mediated through autophagy suppression (34). The contrary effect of the baicalin and celastrol on the autophagy is found.

Silibinin (SB), bisbenzylisoquinoline alkaloids, demethylzeylasteral (T-96), sennoside A (SA), exhibit potent anti-proliferative and anti-migratory effects against PCa cells. Autophagy mechanism is used as the main mechanism to explore SB, bisbenzylisoquinoline alkaloids, T-96, SA in the treatment of PCa. SB significantly inhibits migration and invasion in CRPC through autophagy-mediated downregulation of Yes-associated protein (YAP) (35). It suggests that SB might promote the degradation of YAP in CRPC by activating autophagy. However, whether YAP downregulation by SB involves alternative regulatory pathways, and how YAP functionally interacts with metastatic phenotypes, remain to be elucidated. The bisbenzylisoquinoline alkaloids elicit apoptotic and autophagic cell death in androgen-dependent PCa cells via dual targeting of 5α-reductase and androgen receptor expression through PI3K/AKT pathway suppression (36). T-96 has triggered autophagy via AMPK activation and mTOR inhibition, as evidenced by decreased LC3I levels, reduced p-mTOR, and elevated p-AMPK (37). mTOR inhibition arrests autophagic flux, resulting in autophagosome accumulation rather than autophagy initiation (38). However, the precise role of MAPK/ERK signaling in autophagy activation and maturation remains elusive and contentious.

SA has dose-dependently suppressed viability and induced apoptosis in DU145 and PC-3 cells. SA has activated autophagy, evidenced by elevated LC3B expression, increased LC3-II/I conversion, upregulated beclin-1, and reduced p62 accumulation. SA has inhibited PI3K/AKT/mTOR signaling, as demonstrated by diminished phosphorylation ratios of PI3K, AKT, and mTOR. Pharmacological activation of PI3K using 740Y-P have reversed SA-induced cytotoxicity, apoptosis, and autophagy, confirming pathway specificity (39). These findings establish that SA suppresses proliferation while concurrently inducing apoptosis and autophagy through PI3K/AKT/mTOR pathway inactivation in PCa. Nevertheless, validation using complementary experimental approaches is warranted to substantiate these conclusions.

Autophagy maintains homeostasis in eukaryotic cells by providing energy and a sustainable source of biomolecules under stressful conditions, such as hypoxia, oxidative stress, ER stress, or tumor microenvironment (40). Modifying autophagy through its induction or inhibition is a promising and innovative approach to control cancer progression. It is still a mystery whether autophagy should be considered as a cell death or cell survival response (23). Autophagy may play either a detrimental or a protective role in PCa (15). Autophagy inhibition suppresses proliferation, metastasis, and invasion in various malignancies, while autophagy promotion yields opposite effects (41–43). Starvation triggers autophagy to prevent cells from dying and that if the autophagic pathway is impaired, then cells are led to apoptosis, whereas autophagy may serve as an anticancer mechanism. In the early stages of tumor development, promoting autophagy exerts a tumor-suppressive function by eliminating damaged organelles, reducing oxidative stress, maintaining genomic stability, and degrading oncogenic proteins such as p62/SQSTM1, thereby preventing malignant transformation. However, once tumors are established, cancer cells exploit autophagy as a survival mechanism to adapt to metabolic stress, hypoxia, and nutrient deprivation within the tumor microenvironment; in this context, inhibiting autophagy can impair tumor cell viability, overcome therapeutic resistance, and enhance the efficacy of chemotherapy or radiotherapy. Therefore, the net effect of autophagy modulation—whether promotion or inhibition—on tumor development and patient outcomes depends critically on tumor stage, genetic background, and the specific therapeutic context, rendering autophagy a double-edged sword in oncology. Thus, targeting autophagy to increase the therapeutic effectiveness of anticancer drugs is an innovative strategy for treating cancer.

**Figure 3:**
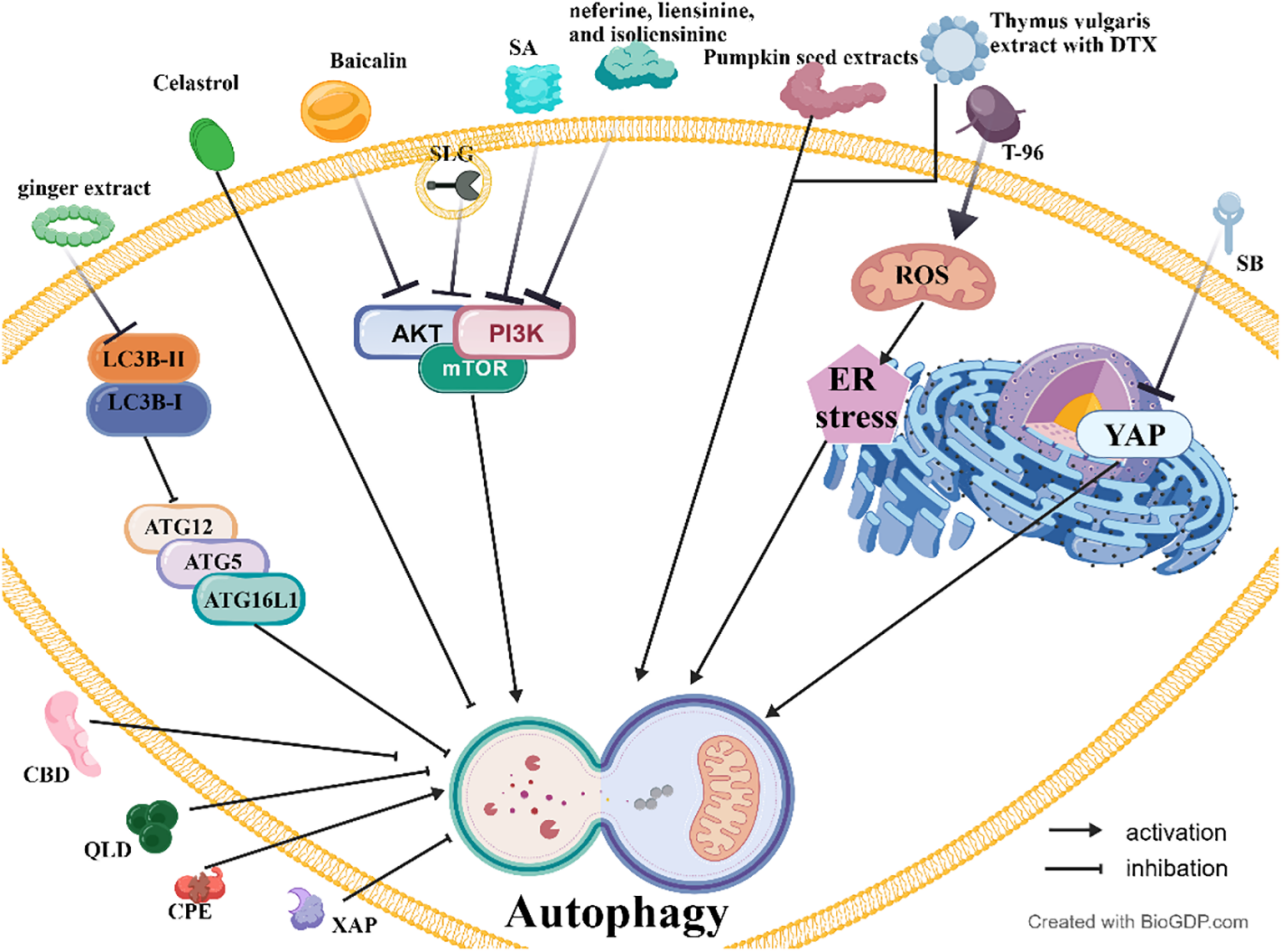
Autophagy of TPM in PCa. Autophagy is enhanced by pumpkin seed extracts, *thymus vulgaris* extract, CP, SLG extract, baicalin, bisbenzylisoquinoline alkaloids, T96, and SA through reducing PI3K/Akt/mTOR and promoting ROS. TPM inhibiting autophagy is inhibited by ginger extract, CBD, XAP Injection, QLD, and celastrol through reducing LC3-II/LC3-I and Atg.

In summary, this section summarizes the regulation of autophagy by TPM in PCa (**Figure 3**). TPM enhancing autophagy include pumpkin seed extracts, thymus vulgaris extract, CP, SLG extract, baicalin, bisbenzylisoquinoline alkaloids, T-96, and SA (**Table 1**). TPM inhibiting autophagy are ginger extract, CBD, XAP Injection, QLD, and celastrol. Autophagy experiment mainly involves exploring its role as a core mechanism, with PCa cell models (PC-3, 22Rv1, LNCaP, DU145, CRPC cell lines). The main mechanisms of autophagy in TPM anticancer effects include regulating PI3K/AKT/mTOR, AMPK/mTOR pathways, ROS levels, and 5-α-reductase/AR expression, with LC3-I/LC3-II ratio, Beclin-1, P62, Atg5/Atg12, YAP, p-mTOR, p-AMPK, p-PI3K, p-AKT. The main deficiencies of current studies are the unclear causes of opposite autophagic effects induced by different TPM and the insufficient exploration of related molecular mechanism. For future research, efforts should focus on clarifying the mechanisms underlying the differential autophagic effects of TPM through *in vivo* experiments in PCa.

**Table 1:** The autophagy in PCa.

Active ingredients	Herbal	Real modules	Study type	Quality indicators	Dose	Pathway of action	Effects/mechanisms	Ref.
Ginger extract	Ginger	PC-3 cells	*in vitro*	3 independent replicates, 3 wells each	12.5–200 μg/mL	LC3B↓, ATG12↓	Suppressing autophagy	(22)
Pumpkin seed extracts	Pumpkin seed	PC-3 cells	*in vitro*	3 independent replicates, 3 wells each	10–200 μg/mL	PC-3 cell viability↓, oxidative parameters↑	Promoting autophagy	(23)
*Thymus vulgaris* extract with DTX	Thymus vulgaris	PC-3 cells	*in vitro*	3 independent replicates, 3 wells each	12.5–200 μg/mL	G0/G1 arrest↑, Apoptosis↑, Autophagy↑	Promoting autophagy	(24)
Cannabidiol (CBD)	Cannabis sativa	PCa cells	*in vitro*	3 independent replicates, 3 wells each	5–20 μg/mL	Autophagic flux↓, ERAD↑, UPR↑	Suppressing autophagy	(4)
*Calotropis procera* extract (CPE)	Calotropis procera	PC-3, 22Rv1 cells	*in vitro*	3 independent replicates, 3 wells each	25–400 μg/mL	Autophagy markers↑	Suppressing autophagy	(25)
*Styrax liquidus* gum (SLG)	Styrax	PC-3, DU145	*in vitro*	3 independent replicates, 3 wells each	25–100 μg/mL	PI3K/Akt/mTOR↓, Autophagy↑	Promoting autophagy	(29)
Xiaoaiping (XAP)	Marsdenia tenacissima	LNCaP, DU145, C4-2, PC-3	*in vitro*	3 independent replicates, 3 wells each	10–320 μg/mL	FoxO3a autophagic degradation↓, Autophagy↓	Suppressing autophagy	(18)
Qi Ling decoction (QLD)	Solanum septemlobum Bunge etc	PC-3, DU145	*in vitro*	3 independent replicates, 3 wells each	10 μg/mL	Apoptosis↑, Cell death↑	Suppressing autophagy	(32)
Silibinin (SB)	Milk thistle seeds	CRPC cells	*in vitro*	3 independent replicates, 3 wells each	25–200 μg/mL	Autophagy↑, YAP degradation↑, Migration↓, Invasion↓, EMT↓	Promoting autophagy	(35)
Bisbenzylisoquinoline alkaloids	Seed-embryos of Nelumbo nucifera Gaertn	PC-3, DU145, LNCaP	*in vitro*	3 independent replicates, 3 wells each	1–100 μg/mL	PI3K/Akt↓, LC3B II↑	Promoting autophagy	(36)
Demethylzeylasteral (T-96)	Tripterygium wilfordii Hook F	PCa cells	*in vitro*	3 independent replicates, 3 wells each	2.5–40 μg/mL	S−phase cell cycle arrest↑	Promoting autophagy	(37)
Sennoside A (SA)	Rheum officinale Baill	Xenografted mice, DU145/PC-3 cells	*in vitro+in vivo*	3 independent replicates (*in vitro*); 6–8 mice/group, 2 experiments (*in vivo*)	50–300 μg/mL	PI3K/AKT/mTOR↓, Autophagic death↑	Promoting autophagy	(39)

↑ = upregulated, ↓ = downregulated.

## Apoptosis

Apoptosis constitutes an energy-dependent, non-inflammatory mode of PCD essential for developmental processes, immune homeostasis, and tumor surveillance (17). The apoptotic pathway is mainly initiated by the death receptor-mediated extrinsic pathway and the Bcl-mediated intrinsic pathway. The combination of Cyt C, Apaf-1, and caspase-9 activates effector caspase-3/7 to execute cell death (44). PCa progression is characterized by apoptotic evasion via anti-apoptotic protein overexpression and p53 inactivation, conferring therapeutic resistance. Conversely, TPM constituents reactivate cell death programs through mitochondrial, endoplasmic reticulum, and death receptor-mediated apoptotic pathways (6). Thus, apoptosis is the target of TPM in the treatment of PCa.

### Plant extract

Apoptosis has been investigated in the context of *Thuja occidentalis hydroalcoholic* extract, *Fagonia cretica*, and *mistletoe* extracts. *T. occidentalis hydroalcoholic* extract has anti-proliferative, anti-metastatic and pro-apoptotic effects, and it presents cytotoxic effects on PCa cell lines and androgen unresponsive cells through apoptosis, necrosis, and cell cycle arrest (45). *Fagonia cretica* L., a plant with traditional medicinal use in oncology, elicits significant DNMT1 downregulation concurrent with apoptotic induction and oxidative stress generation (46). *Annona crassiflora* Mart. (araticum), a native fruit of the Brazilian Cerrado, contains bioactive constituents with demonstrated antineoplastic properties. Notably, its seed extract potently induces apoptosis in LNCaP PCa cells (47). Mistletoe (*Viscum album L.*) extracts, widely utilized as complementary cancer therapeutics, exert antiproliferative effects through G2/M cell-cycle arrest and apoptotic induction in Populus and Salix-derived preparations (48). These findings indicate apoptosis in PCa treatment. However, apoptosis is not the primary focus. Future investigations should focus on isolating bioactive constituents and validating therapeutic efficacy in preclinical models, thereby substantiating the ethnomedicinal application of these plant extracts as antineoplastic agents.

The following studies have specifically investigated the apoptotic mechanism of the patchouli alcohol*, Buxus natalensis*, and *olive* leaf extracts. *Pogostemonis* Herba is abundant in patchouli alcohol, which exhibits antioxidant, anti-inflammatory, and anticancer effects (49). Plumbagin (PA) suppresses proliferation, migration, and invasion in CRPC cells, while concurrently inducing mitochondrial apoptosis via NF-κB/Mcl-1 inhibition. Notably, Mcl-1 overexpression attenuates PA-induced cytotoxicity, confirming pathway dependency (50). In the same lab, network pharmacology analysis has been used in the effect of PA on PCa. The effects of GPBAR1 overexpression and silencing regulate cell proliferation, apoptosis, migration and invasion of PCa. PA has regulated the GPBAR1/NF-κB pathway (51). These experiments reveal that PA inhibits the migration, growth, and invasion of CRPC cells *in vitro* and PCa *in vivo* by inducing apoptosis. The mitochondrial apoptosis pathway and GPBAR1/NF-κB pathway regulate the role of PA in PCa treatment. The overexpression and silencing of Mcl-1 and GPBAR1 have verified the conclusion. Bidirectional validation has been performed to unequivocally establish the essential contributions of the two pathways. It further confirms a novel biomarker for PA in the treatment of PCa. It is innovative to explore the difference between NF-κB/Mcl-1 pathway and GPBAR1/NF-κB pathway. *Buxus natalensis*, a rich source of antineoplastic triterpenoidal alkaloids, yields hydroethanolic leaf extracts (BNHLE) that exert potent cytotoxicity against malignant cell lines. BNHLE activated apoptosis across malignant cell lines through p53 upregulation coupled with NF-κB-p65 and Bcl-2 suppression, as evidenced by elevated Bax/Bcl-2 ratios confirming commitment to PCD (52). These findings support the ethnobotanical utilization of *Buxus natalensis* as a promising reservoir of antineoplastic agents against PCa. Olive (*Olea europaea* L.) leaf and pomace residues constitute valuable sources of bioactive phenolics with demonstrated antioxidant and antineoplastic properties. Pomace extracts have suppressed PC-3 cell proliferation, elicited morphological and phenotypic alterations, perturbed cell-cycle dynamics, and promoted apoptotic nuclear morphogenesis, ultimately triggering PCD (53). Collectively, these results highlight olive products as valuable and promising sources of natural anticancer compounds for pharmaceutical development.

### Chinese herbal compound

Apoptosis mechanism is used as the main mechanism to explore in the TPM of *Hedyotis diffusa* Herba *- Scutellaria barbata* Herba (HDH-SBH) and *Zhoushi Qiling decoction* (*ZQD*). The drug pair Hedyotis Diffusae Herba (HDH) and Scutellaria Barbatae Herba (SBH)-a classic antitumor combination in TCM. The HDH-SBH shows significant antitumor value. HDH-SBH constitutes a classic herbal combination with potent antitumor activity in traditional Chinese medicine. This herb pair exerts remarkable inhibitory effects against tumor progression. *In vitro*, HDH-SBH induces significant apoptosis through suppressing Bcl2 and p-65 protein expression, AKT1 phosphorylation (54). Thus, HDH-SBH suppresses PCa progression by regulating apoptosis.

A limitation of this study is that it only focuses on a single androgen-independent cell line. Subsequent research will benefit from incorporating both androgen-sensitive and normal prostate cell lines to more comprehensively evaluate HDH-SBH’s therapeutic and range of efficacy. ZQD demonstrates significant potential in alleviating discomfort associated with endocrine therapy and extending survival rates among PCa patients. ZQD treatment promoted apoptosis of PCa cells through the upregulation of miR-143, which elucidates a previously unrecognized mechanism underlying its inhibitory effect on PCa (55). However, small sample sizes and the lack of placebo controls warrant further validation in the efficacy of ZQD for PCa therapy.

### Pure compound

Apoptosis is mentioned in the *Tripterygium wilfordii* Hook. f. (TW), kerstinginone, xanthatin, and *3-*acetyl-11-keto-beta-boswellic acid (AKBA). TW is extensively utilized in clinical practice for its effective anti-inflammatory and anti-cancer properties. Triptonoterpene of TW has subsequently been confirmed to induce apoptosis (56). Kerstinginone is a chemical constituent isolated from the stem barks and leaves of *Commiphora kerstingii*, and exhibits apoptosis-inducing activity (57). Xanthatin is extracted from *Xanthium strumarium* L., and induces apoptosis by the promotion of caspase-3 (58). AKBA is a monomer extracted from the *Boswellia* that has induced cell apoptosis (59). These experiments demonstrate potential effectiveness of apoptosis in PCa treatment and present a novel direction for developing natural compound-based anti-cancer therapies.

Butein and puerarin (PEU) exhibit potent antimigratory effects against PCa cells. Apoptosis mechanism is used as the main mechanism to explore in the butein and PEU. Butein increased Bax, decreased Bcl-2, caspase-3, PARP, p-receptor interacting serine/threonine-protein kinase 3 (p-RIP3) and p-mixed lineage kinase domain-like kinase (p-MLKL) (60). It indicates that necrosis and apoptosis are induced by butein in PCa cells by ROS. Different sensitivities are observed in PC-3 cells and DU145 cells, and such differences will be evaluated in the *in vivo* animals. PEU induces apoptosis in PC-3 and DU145 cells by upregulating Bax and cleaved caspase-3, and reduction of Bcl-2. Keap1 protein expression is increased, and Nrf2, HO-1 and NQO1 protein expression are decreased in PC-3 and DU145 cells by PEU (61). These findings indicates that the Keap1/Nrf2/ARE signaling pathway is the target of the apoptosis in DU145 and PC-3 cells by PEU.

**Figure 4:**
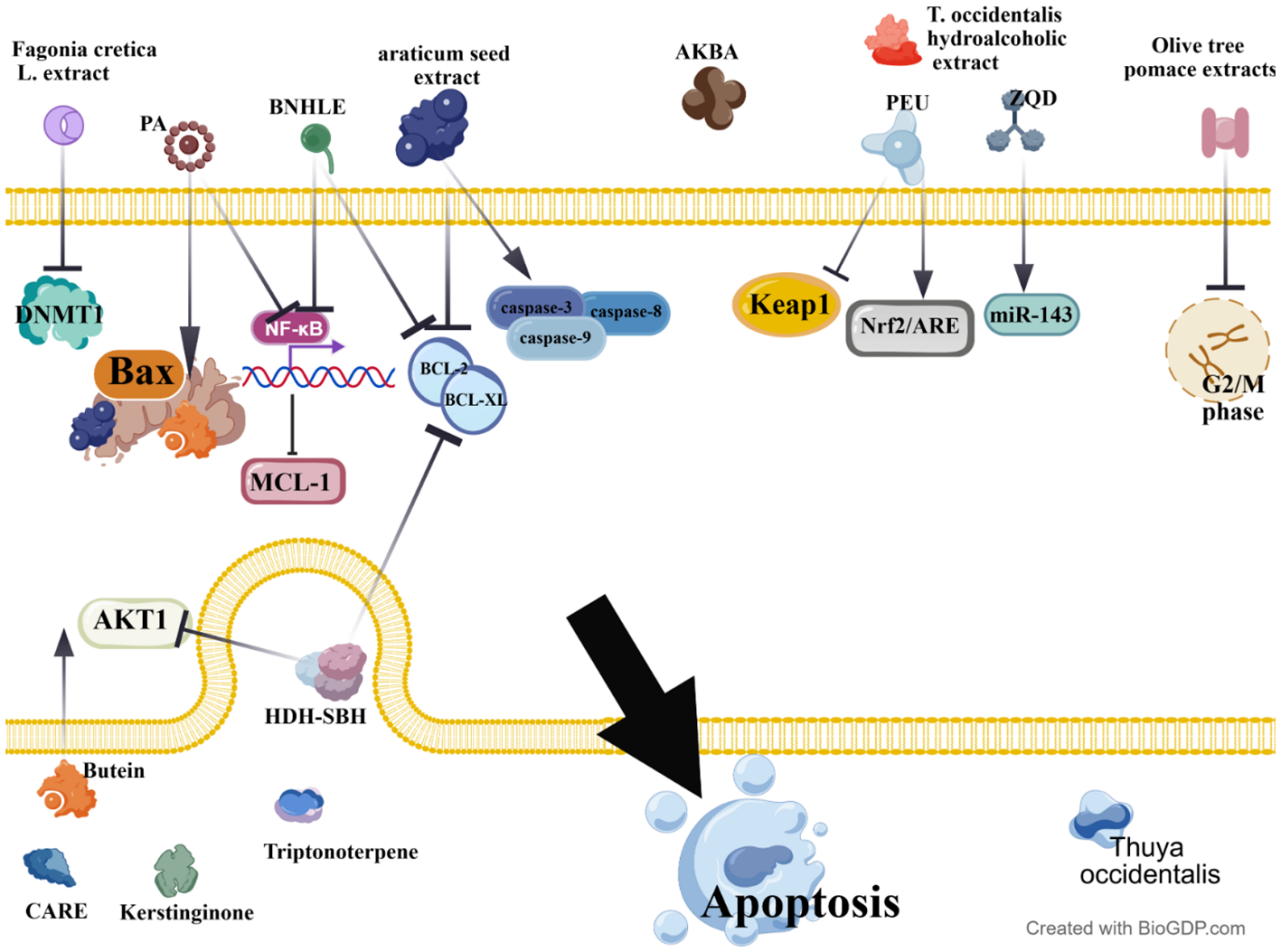
Apoptosis of TPM in PCa. PA, Bax, caspase 3, 8, 9, Nrf2/Are, mir-143, Akt1 are increased by *Araticum* seed extract, AKBA, PEU, ZQD, *T.occidentalis* hydroalcoholic, CARE, BUTEIN, triptonoterpene, *thuya occidentails*, inducing apoptosis. DNMT1, NF-KB, Bcl-2, Bcl-XL, Keap1, G2/Mphase, and AKT1 are reduced by *Fagonia cretica* L. extract, PA, BNHLE, *Araticum* seed extract, PEU, Olive tree pomace extracts, and HDH-SBH.

In conclusion, this section summarizes the regulation of apoptosis by TPM in PCa (**Figure 4**). No TPM inhibits apoptosis; most TPM induce it, including plant extracts (T. *occidentalis hydroalcoholic* extract, *Fagonia cretica, araticum* seed extract, patchouli alcohol, *Buxus natalensis* extract, olive pomace extract, Chinese herbal compounds (HDH-SBH, ZQD), and pure compounds (triptonoterpene of TW, kerstinginone, xanthatin, AKBA, butein, PEU) (**Table 2**). Apoptosis experiment focus on its core regulatory role, with models including PCa cell lines (PC-3, DU145, LNCaP, CRPC) and small clinical samples. Main mechanisms involve mitochondrial pathway, endoplasmic reticulum stress, death receptor signaling, and key pathways (AKT1/Bcl2/NFKB, GPBAR1/NF-κB, Keap1/Nrf2/ARE), via regulating pro/anti-apoptotic proteins and ROS. Key indicators inlcude p53, Bcl-2, Bax, caspase-3, PARP, NF-κB-p65, Keap1, Nrf2, Mcl-1, DNMT1. An innovative aspect is the exploration of associations among key factors in the apoptosis mechanism of PCa. The TPM offers promise as a natural therapeutic candidate for PCa management.

**Table 2:** The apoptosis in PCa.

Active ingredients	Herbal	Real modules	Study type	Quality indicators	Dose	Pathway of action	Effects/mechanisms	Ref.
T.occidentalis hydroalcoholic extract	T. occidentalis	PCa cells	in vitro	3 independent replicates, 3 wells each	0.05 mL (volume)	Apoptosis↑, Necrosis↑, Cell cycle arrest↑	Inducing apoptosis	(45)
Fagonia cretica L. extract	Fagonia cretica L.	PC-3 cells	in vitro	3 independent replicates, 3 wells each	20 μg/mL	DNMT1 protein↓, DNMT1 mRNA↓	Inducing apoptosis	(46)
Araticum seed extract	Annona crassiflora Mart.	PC-3, LNCaP, 22Rv1	in vitro	3 independent replicates, 3 wells each	5–320 μg/mL	Bax↑, procaspase 3↑, caspase 9↑, caspase 8↑, Bcl-2↓, Bcl-xL↓	Inducing apoptosis	(47)
Populus and Salix	Plant-derived mistletoe	PC-3, DU145, LNCaP	in vitro	3 independent replicates, 3 wells each	1:20–1:160000 (dilution)	G2/M phase cell-cycle arrest↑	Inducing apoptosis	(48)
Patchouli alcohol (PA)	Pogostemonis Herba	PC-3, DU145 cells	in vitro	3 independent replicates, 3 wells each	50–100 μg/mL	GPBAR1/NF-κB/PI3K/AKT↓	Inducing apoptosis	(49–51)
Buxus natalensis natalensis leaf extract (BNHLE)	Buxus natalensis	PC-3, DU145	in vitro	3 independent replicates, 3 wells each	10–500 μg/mL	p53↑, NF−κB−p65↓, Bcl−2↓	Inducing apoptosis	(52)
Olive tree pomace extracts	Olive tree	PC-3, MDA-MB-231	in vitro	3 independent replicates, 3 wells each	50–400 μg/mL	Proliferation↓, Morphological alterations↑, Cell cycle modification↑, Apoptotic nuclear changes↑	Inducing apoptosis	(53)
Hedyotis Diffusae Herba-Scutellariae Barbatae Herba (HDH-SBH)	Hedyotis diffusa Herba, Scutellaria barbata Herba	PC-3 cells	in vitro	3 independent replicates, 3 wells each	0.25–2.0 mg/mL	Bcl2↓, p−65↓, AKT1 phosphorylation↓	Inducing apoptosis	(54)
Zhoushi Qiling decoction (ZQD)	Radix Astragal, Herba Epimedii etc	PC-3, DU145	in vitro	3 independent replicates, 3 wells each	12.5–400 μg/mL	miR−143 expression↑	Inducing apoptosis	(55)
Triptonoterpene	Tripterygium wilfordii Hook. f.	PC-3, DU145, LNCaP	in vitro	3 independent replicates, 3 wells each	0.1–20 μg/mL	PCa cell growth↓, Apoptosis↑	Inducing apoptosis	(56)
Kerstinginone	Commiphora kerstingii Engl	PC-3, LNCaP	in vitro	3 independent replicates, 3 wells each	2.5–10 μg/mL	Cytotoxicity↑	Inducing apoptosis	(57)
Xanthatin	Xanthium strumarium L.	DU145 cells	in vitro	3 independent replicates, 3 wells each	2.5–40 μg/mL	Caspase−3↑, CDK6↓	Inducing apoptosis	(58)
3-acetyl-11-keto-beta-boswellic acid (AKBA)	Boswellia	DU145 cells	in vitro	3 independent replicates, 3 wells each	10–40 μg/mL	Proliferation↓, Invasion↓, Metastasis↓	Inducing apoptosis	(59)
Butein	Rhus verniciflua Stokes	PC-3, LNCaP	in vitro	3 independent replicates, 3 wells each	10–30 μg/mL	p−RIPK3↑, p−MLKL↑	Inducing apoptosis & necroptosis	(60)
Puerarin (PEU)	Pueraria lobata	PC-3, DU145, LNCaP	in vitro	3 independent replicates, 3 wells each	2.5–10 μM	Keap1/Nrf2/ARE signaling↓	Inducing apoptosis	(61)
Curcuma amada rhizome extract (CARE)	Curcuma amada	PC-3 cells	in vitro	3 independent replicates, 3 wells each	10–200 μg/mL	Proliferation↓, PI3K−AKT signaling↓	Inducing apoptosis	(62)

↑ = upregulated, ↓ = downregulated.

## Pyroptosis

Pyroptosis is a type of PCD that is inherently pro-inflammatory and dependent on inflammasome activation (17). The pyroptotic pathway can be divided into the NLRP3-induced canonical pathway and the LPS-induced noncanonical pathway, which activates caspase-1 and GSDMD to cause the release of IL-18, IL-1β, and DAMPs. Chemotherapies induce caspase-3-mediated GSDME cleavage to cleave cells and release DAMPs (44). Apoptosis is a classic therapeutic target, and pyroptosis offers new strategies for apoptosis-resistant tumors. Pyroptosis suppresses the metastasis of cancer cells (63, 64). Pyroptosis is crucial in PCa because its ability to regulate invasion, proliferation, and metastasis offers significant therapeutic potential, a relevance that extends to several other malignancies as well. *Astragaloside* IV is extracted from *Astragalus*, and exhibits antitumor, antioxidative, and anti-inflammatory activities. The palmitoylation modification of the GSDMD and the upregulation of ZDHHC1 expression are facilitated by *astragaloside* IV, promoting pyroptosis in PCa cells (64). It indicates that ZDHHC1 plays a physiological role in pyroptosis, providing additional molecular insights into the pyroptosis mechanism of TPM in PCa.

Pyroptosis mechanism is used as the main mechanism to explore in the treatment of calycosin and icariin plus curcumol in PCa. Calycosin inhibits pyroptosis and improves mitochondrial damage in heart failure through the Nrf2/ROS/TXNIP pathway, which may disrupt the crosstalk between mitochondrial damage and pyroptosis, thereby exerting cardioprotective effects (65). Calycosin substantially ameliorated the pathological damage of prostate tissue. Calycosin has remarkably reduced the inflammatory factors secretion and oxidative stress markers. Calycosin effectively suppresses pyroptosis, which is linked with inactivation of the NF-κB-p65 signaling pathway. These beneficial effects of Calycosin are similar to those of the NLRP3 inhibitor. It indicates that Calycosin protects against Chronic prostatitis (CP) via suppressing inflammation, oxidative stress, and pyroptosis through NF-kBp65 pathway, thereby a promising therapeutic option for CP (66). Silencing Nrf2 partially reversed the cardioprotective effects of CA, confirming the key therapeutic role of Calycosin in Nrf2-mediated anti-pyroptosis (65). Further research on the PCa model is warranted. Icariin plus curcumol demonstrates great potential as a therapeutic agent for CRPC by enhancing autophagy *via* the mTOR pathway and promoting pyroptosis mediated by cathepsin B (67). These findings provide valuable insights into the molecular mechanisms underlying the therapeutic potential of icariin and curcumol for PCa. However, the contradiction on the pyroptosis in PCa between the calycosin, Icariin plus curcumol and *astragaloside* IV may be attributed to the different pathologies between chronic prostatitis and CRPC. NF-kB-p65 pathway and the Nrf2/ROS/TXNIP pathway have the inhibition of pyroptosis. The mTOR pathway and cathepsin B have mediated the promotion of autophagy and pyroptosis in Icariin plus curcumol treatment of CRPC (67). However, the NF-κB-p65 pathway and the mTOR pathway exhibit a well-established interactive relationship, modulating each other through multiple mechanisms to form a complex signaling network. The effect of CA on the pyroptosis of PCa needs further research to explore. Pyroptosis plays a regulatory role in tumor cell invasiveness, differentiation, proliferation, and metastasis (68). Pyroptosis is mediated by the gasdermin family, accompanied by inflammatory and immune responses. The dual mechanisms by which pyroptosis-related factors promote and inhibit tumor development remain to be explored. The effects of pyroptosis on cancer vary in different tissues and genetic backgrounds. Pyroptosis can inhibit the occurrence and development of tumors. As a type of proinflammatory death, pyroptosis can form a suitable microenvironment for tumor cell growth and thus promote tumor growth. The induction of tumor pyroptosis is also considered a potential cancer treatment strategy. Therefore, the potential role of pyroptosis in regulating the development of PCa warrants further investigation and attention.

**Figure 5:**
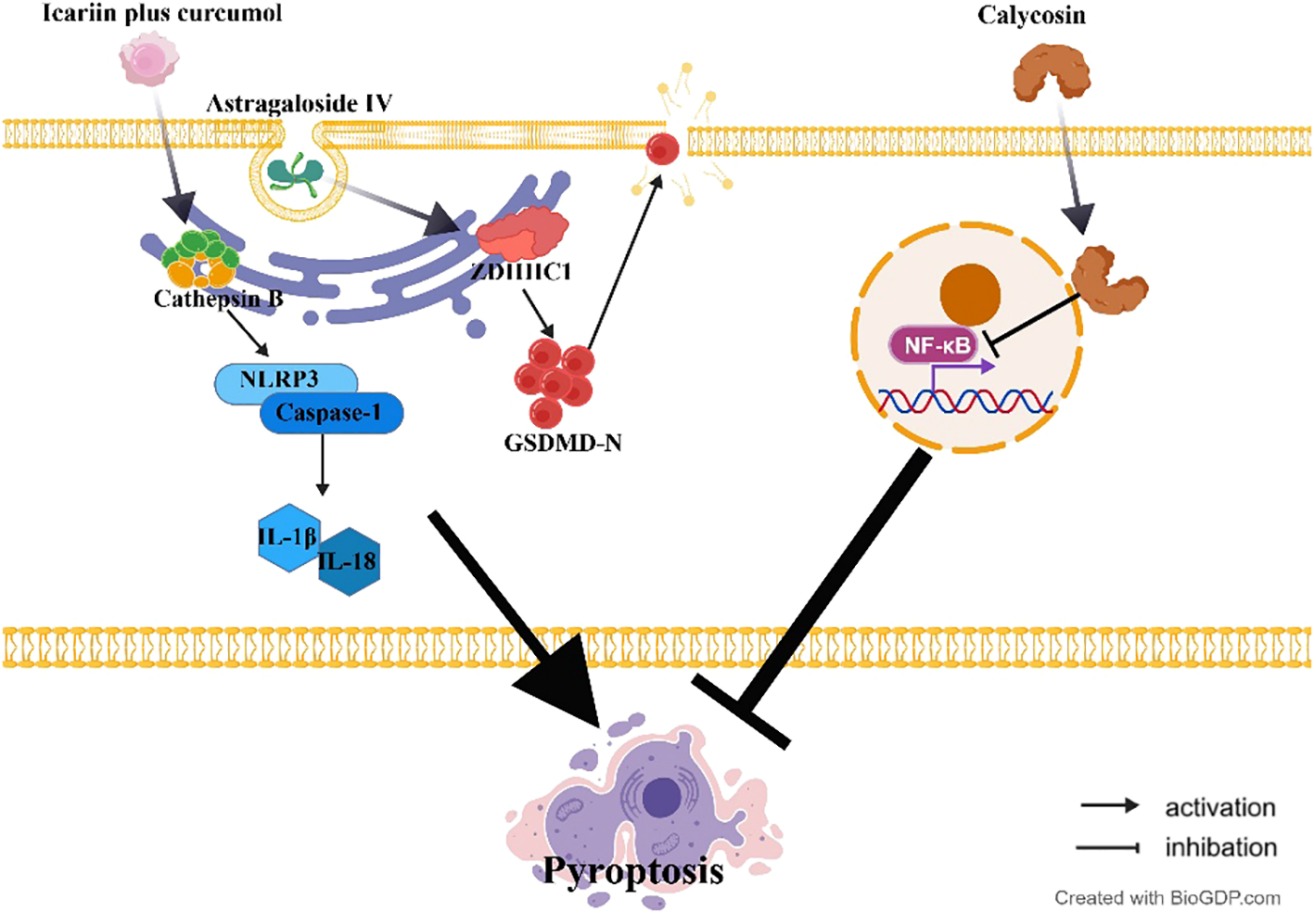
Pyroptosis of TPM in PCa. Icariin plus curcumol increase cathepsin B, NLRP3, Caspase1, promoting the inflammation cytokine of IL-1β and IL-18. Astragaloside IV increase ZDHHC1 and GSDMD-N. Icariin plus curcumol and Astragaloside IV promote pyroptosis. Calycosin increase NF-KB, suppressing pyroptosis.

In conclusion, this section summarizes pyroptosis regulation by TPM in PCa (**Figure 5**). TPM inducing pyroptosis include *Astragaloside* IV and icariin plus curcumol (**Table 3**). Calycosin inhibits pyroptosis in chronic prostatitis, and its effect on PCa pyroptosis is unclear. Pyroptosis experiment focus on its regulatory role in antitumor effects, with models including PCa cell lines (PC-3, CRPC) and prostate tissue (chronic prostatitis model, PCa model pending). Main mechanisms involve regulating GSDMD palmitoylation, NF-κB-p65 pathway, mTOR pathway and cathepsin B. Key indicators include GSDMD, ZDHHC1, NF-κB-p65, cathepsin B, inflammatory factors, oxidative stress markers. Few PCa models for pyroptosis research, unclear effect of calycosin on PCa pyroptosis, insufficient detection of cell death modes other than pyroptosis. This provides references for subsequent pyroptosis-related anti-cancer trial.

**Table 3:** The pyroptosis in PCa.

Active ingredients	Herbal	Real modules	Study type	Quality indicators	Dose	Pathway of action	Effects/mechanisms	Ref.
Astragaloside IV	Astragalus	PC-3, DU145	in vitro	3 independent replicates, 3 wells each	10–40 μg/mL	ZDHHC1↑, GSDMD−N membrane translocation↑	Inducing pyroptosis	(65)
Calycosin	Radix Astragali	RWPE−1 cells	in vitro	3 independent replicates, 3 wells each	10–40 μM	Pyroptosis↓, NF−κBp65 signaling↓	Inhibiting pyroptosis	(67)
Icariin plus curcumol	Ezhu	PC-3 cells	in vitro	3 independent replicates, 3 wells each	20 μM Icariin+40 μM Curcumol	Cathepsin B-mediated pyroptosis↑	Inducing pyroptosis	(68)

↑ = upregulated, ↓ = downregulated.

## Ferroptosis

Ferroptosis is a form of cell death characterized by the accumulation of lipid peroxides in cellular membranes accompanied by iron (Fe²^+^) accumulation in the cellular membranes (17, 69). In recent years, inhibiting tumor growth and overcoming tumor drug resistance by inducing ferroptosis has become a hot research topic. Ferroptosis modulates tumor cell proliferation (63), and dynamic equilibrium of PCa (6). Targeting ferroptotic pathways emerges as an innovative therapeutic avenue for combating advanced prostate malignancies.

### Plant extract

Ferroptosis is mentioned in studies on stachydrine hydrochloride. Stachydrine hydrochloride has reduced paclitaxel resistance, promoted apoptosis, senescence, and ferroptosis pathways in CRPC (70). These findings illuminate the prospective value of stachydrine hydrochloride as an innovative combinatorial agent to circumvent taxane resistance in CRPC. However, the ferroptosis pathway is one of the contents of this experiment, and its mechanism is not deeply explored.

### Chinese herbal compound

Traditional Chinese herbal medicines have exhibited anti-cancer activities. PC-3 cancer cell proliferation and tumor growth have been inhibited by inhibition of QLD on FSP1, which has increased the ferroptosis and the sensitivity of PC-3-AbiR cells to abiraterone (71). This investigation unveils unprecedented mechanistic insights into therapeutic evasion in prostate malignancies, offering iron-dependent cell death as a viable paradigm for refining clinical intervention strategies.

### Pure compound

The induction of ferroptosis in PCa by pure compound has been recognized in artesunate. Artesunate (ART), a semi-synthetic derivative of artemisinin, demonstrates potent antineoplastic efficacy across diverse malignant cell types, including prostate carcinoma. This compound emerges as a viable adjunctive candidate for ferroptosis-targeted interventions in PCa management (72). However, the differential regulatory mechanisms of apoptotic and ferroptotic pathways in different cell types have not been analyzed in-depth.

Berberine, dihydrochelerythrine (DHC), ginsenoside Rh2 (GRh2), and evodiamine exhibits multifaceted pharmacological properties encompassing antioxidant, anti-inflammatory, and antineoplastic activities. Ferroptosis mechanism is used as the main mechanism to explore in the berberine, dihydrochelerythrine (DHC), ginsenoside Rh2 (GRh2), and evodiamine. Ferroptosis is the targets of berberine in PCa through SCD, ICAM1, AURK, and PTGS2 (73). These findings illuminate berberine’s mode of action in prostate malignancies, implicating iron-dependent lipid peroxidation as the principal mechanistic underpinning. However, the study does not verify the specific roles of core hub genes through functional deficiency experiments. In DU145 and PC-3 cells, DHC has triggered robust ferroptotic responses characterized by GPX4 suppression, dense mitochondrial matrices, and cristae vanishing, ultimately driving lipid ROS generation. This cytotoxic effect is substantially attenuated upon ferrostatin-1 co-treatment, affirming the specificity of iron-dependent cell death induction (74). However, subsequent investigations must delineate the underlying signaling cascades to comprehensively elucidate this compound’s therapeutic utility in clinical PCa management.

GRh2 has concurrently instigated mitochondrial impairment—characterized by PINK1/Parkin-mediated mitophagy activation, diminished VDAC1/TOM20 expression, and autophagosome formation—and iron-dependent cell death, evidenced by lipid peroxide accumulation, elevated MDA/Fe²^+^ levels, GSH exhaustion, and SLC7A11/GPX4 suppression. The specificity of these parallel mechanisms is validated through Mdivi-1 and Fer-1 rescue experiments. Notably, GRh2 has significantly attenuated tumor progression in xenograft models (75). GRh2 orchestrates convergent antineoplastic mechanisms in prostate malignancies, simultaneously engaging mitophagy-mediated mitochondrial deterioration and iron-dependent lipid peroxidation to achieve synergistic tumor suppression.

Evodiamine has elicited robust oxidative stress responses. The causative involvement of ROS in this ferroptosis cascade is corroborated by N-acetylcysteine-mediated partial rescue of cytotoxicity. Mechanistically, evodiamine has instigated iron-dependent cell death through TRIM26-mediated GPX4 proteolysis. Substantial tumor growth suppression and ferroptosis induction have been observed in xenograft models, underscoring the translational viability of this alkaloid for prostate malignancy intervention (76). These findings establish evodiamine as a potent ferroptosis inducer in prostate malignancies, wherein disruption of TRIM26-dependent GPX4 maintenance serves as the primary molecular conduit for its antineoplastic efficacy.

**Figure 6:**
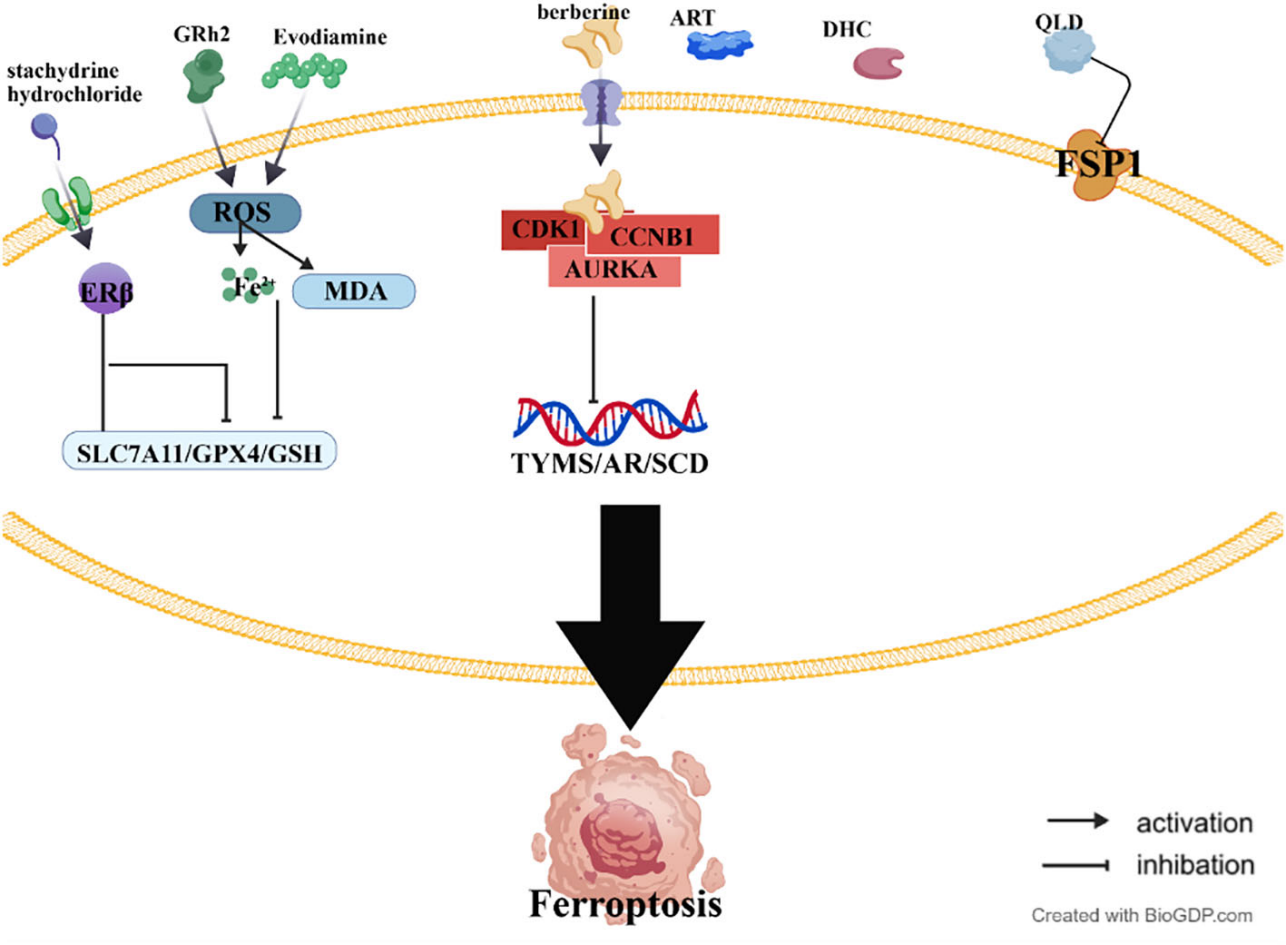
Ferroptosis of TPM in PCa. ERβ, ROS, CDK1, CCNB1, AURKA, FSP1 are increased by stachydrine hydrochloride, GRh2, evodiamine, berberine, ART, DHC and QLD, promoting ferroptosis.

In conclusion, this section also summarizes ferroptosis regulation by TPM in PCa (**Figure 6**). TPM that induce ferroptosis include plant extracts (stachydrine hydrochloride), QLD, and pure compounds (artesunate, berberine, DHC, GRh2, evodiamine) (**Table 4**). No TPM inhibits ferroptosis. Ferroptosis experiment focus on its role in overcoming drug resistance and antitumor effects, with models including PCa cell lines (PC-3, DU145, PC-3-TxR, PC-3-AbiR) and *in vivo* tumor models. Main mechanisms involve inhibiting FSP1/GPX4/TRIM26, regulating lipid ROS and iron metabolism, and core pathways related to TYMS, AR, and PINK1/Parkin. Key indicators include Fe^2+^, lipid peroxides, MDA, GSH, GPX4, FSP1, SLC7A11, ROS, and TRIM26. Ferroptosis is usually detected with apoptosis, senescence or mitophagy, but other cell death modes are rarely detected. Further studies need to verify the specific roles of core hub genes through functional deficiency experiments. The key role of ROS is confirmed in evodiamine-induced ferroptosis by NAC.

**Table 4:** The ferroptosis in PCa.

Active ingredients	Herbal	Real modules	Study type	Quality indicators	Dose	Pathway of action	Effects/mechanisms	Ref.
Stachydrine hydrochloride	Leonurus japonicus	PC-3, DU145, PC-3-AbiR	*in vitro*	3 independent replicates, 3 wells each	100–150 μM	Estrogen receptor beta↑	Inducing ferroptosis	(71)
Qi Ling decoction (QLD)	Astragalus membranaceus, Curcuma longa, Rehmannia glutinosa, etc	PC-3, DU145, PC-3-AbiR	*in vitro*	3 independent replicates, 3 wells each	2% QLD-containing serum	Sensitivity to abiraterone↑, FSP1↓	Promoting ferroptosis	(72)
Artesunate (ART)	Artemisia annua	PC-3, DU145, LNCaP, TEC	*in vitro*	3 independent replicates, 3 wells each	1–100 μM	Cell cycle arrest↑, Apoptosis↑	Inducing ferroptosis	(73)
Berberine	Coptis chinensis Franch.	PC-3, DU145	*in vitro*	3 independent replicates, 3 wells each	5 μmol/L	Binding to 10 core genes↑, ferroptosis-related genes↓	Inducing ferroptosis	(74)
Dihydrochelerythrine (DHC)	Corydalis yanhusuo	PC-3, DU145	*in vitro*	3 independent replicates, 3 wells each	5 μM, 10 μM	GPX4↓, mitochondrial density↑, cristae↓	Inducing ferroptosis	(75)
Ginsenoside Rh2 (GRh2)	Panax ginseng	PC-3 cells	*in vitro*	3 independent replicates, 3 wells each	19.3 μg/mL	Lipid ROS↑, MDA/Fe²^+^↑, GSH↓, SLC7A11/GPX4↓	Inducing ferroptosis	(76)
Evodiamine	Evodia rutaecarpa	Mouse xenograft (DU145 derived)	*in vitro+in vivo*	3 independent repeats (*in vitro*); 6–8 mice/group, 2 experiments (*in vivo*)	0.5–5.0 μM (*in vitro*); [dose not specified]	ROS↑, Lipid ROS↑, MDA↑, GSH↓	Inducing ferroptosis	(77)

↑ = upregulated, ↓ = downregulated.

## Necroptosis

Necroptosis represents a regulated form of necrotic cell death marked by mitochondrial dysfunction, plasma membrane rupture, and the elicitation of inflammatory responses (77). The necroptotic pathway is initiated when caspase-8 is absent by RIPK1-mediated RIPK3 activation. MLKL is then recruited and phosphorylated to form pores in the membrane and the release of DAMPs (44). Notably, necroptosis exerts decisive modulatory roles in PCa drug responsiveness. The necrosome forms through RIP1-RIP3 mutual phosphorylation, initiating programmed necroptosis. Notably, in acidosis-conditioned PCa cells, apoptosis and necroptosis are concurrently activated, with this dual cell death response driven by oxidative stress and mitochondrial dysfunction (78). These findings illuminate the dual nature of TPM in necroptosis-targeted therapeutics.

Curcumin, 20(S)-ginsenoside Rg3 (20(S)-Rg3), and Shikonin (SHI) are isolated from *Curcuma longa rhizomes*, Panax ginseng, and *Lithospermum erythrorhizon.* They have exhibited broad-spectrum of antitumor activity. Curcumin inhibits cell proliferation, induces apoptosis and necroptosis, suppresses angiogenesis and epithelial–mesenchymal transition, and enhances the susceptibility of malignant cells to chemotherapy and radiotherapy (79). 20(S)-Rg3 inhibits the proliferation of PCa cells *in vitro* and upregulates the expression of necroptotic proteins, and pretreatment with the selective RIPK1 inhibitor necrostatin-1 has partially reversed the inhibitory effect of 20(S)-Rg3 on PCa cell proliferation (80). These findings indicates that 20(S)-Rg3 might induce necroptosis in PCa *in vitro* via the ROS/autophagy signaling pathway. *In vivo*, however, no noteworthy differences in tumor size were observed between control and treated mice, possibly due to the short treatment time. These findings suggest that 20(S)-Rg3 induces necroptosis *in vitro*, but its *in vivo* antitumor efficacy requires further validation with optimized dosing regimens.

Shikonin (SHI) has concurrently triggered apoptotic and necroptotic cascades in parental and docetaxel-resistant prostate carcinoma cells, as evidenced by elevated RIP1/RIP3 phosphorylation. RIP1 and RIP3 constitute pivotal mediators of necroptotic execution. Concordantly, S166 phosphorylation of RIP1, an established hallmark of necrosome activation, is readily detectable in naive PC-3 and DU145 cells. The necroptotic specificity of this cytotoxic response is corroborated through necrostatin-1-mediated partial rescue of proliferative capacity (81). The engagement of necroptotic pathways by SHI has been hypothesized as a strategic mechanism to circumvent apoptotic evasion. Consistent with this premise, SHI-directed programmed necrosis has effectively precluded tumor cell escape, culminating in substantial growth suppression of prostate malignancies. Despite these molecular hallmarks of necroptosis, none of the parental or DX-resistant prostate carcinoma cell lines displayed obvious necrotic morphological features following treatment. Accordingly, unprogrammed necrosis cannot explain the antiproliferative effect exerted by SHI.

**Figure 7:**
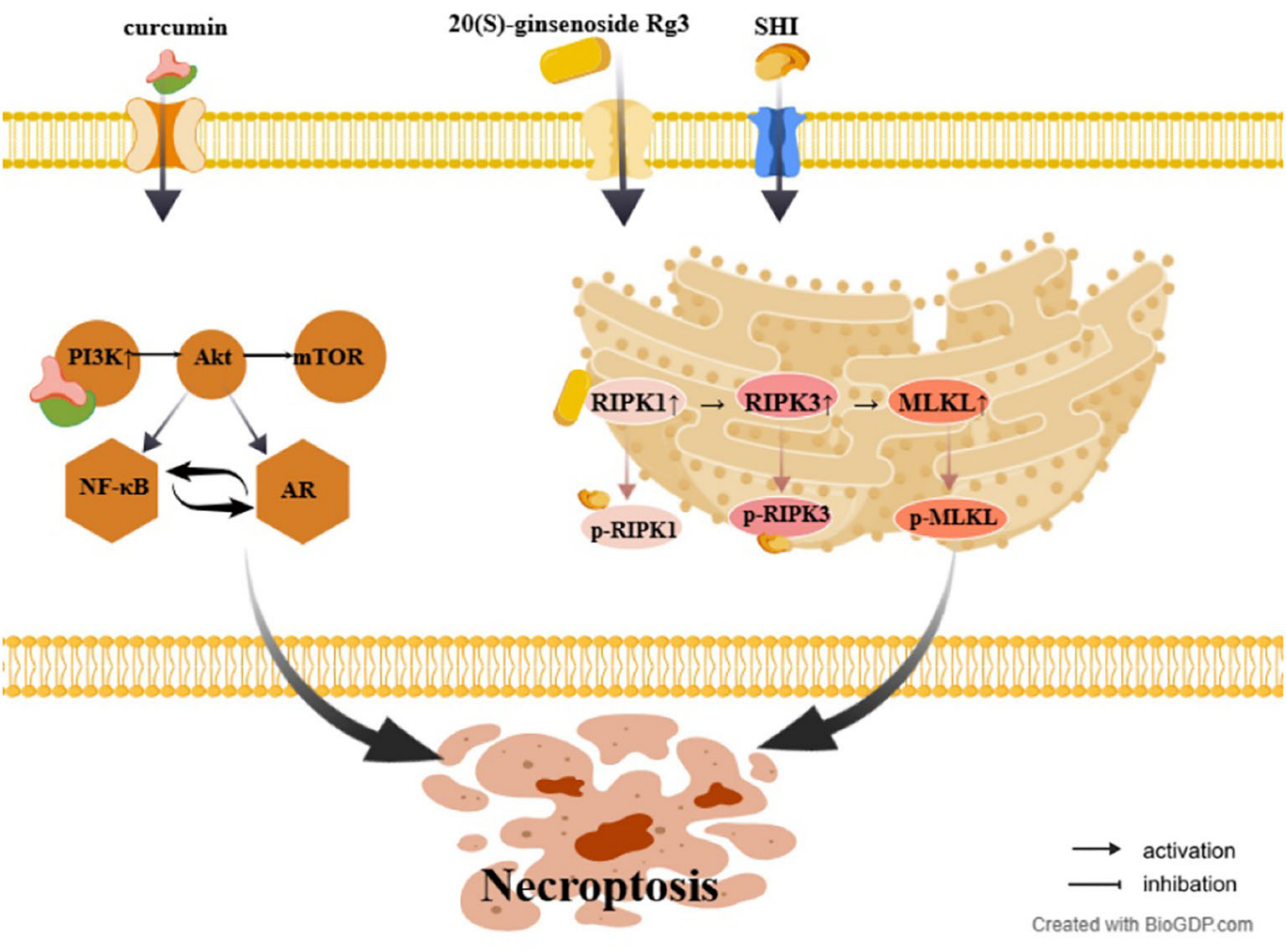
Necroptosis of TPM in PCa. PI3K/Akt, RIPK, and MLKL are increased by curcumin, 20(S)-Rg3, and SHI, promoting necroptosis.

In conclusion, this review also summarizes necroptosis regulation by TPM in PCa (**Figure 7**). TPM that induce necroptosis include curcumin, 20(S)-Rg3, and SHI (**Table 5**). No TPM inhibits necroptosis. Necroptosis experiment focus on overcoming drug resistance and exerting antitumor effects, with models including PCa cell lines (PC-3, DU145, DX-resistant PCa cells) and *in vivo* mouse models. Main mechanisms involve activating RIP1/RIP3 to form the necrosome, regulating ROS/autophagy signaling pathway, and upregulating p62, with necroptosis inhibitors (necrostatin-1) used for verification. Key indicators include RIP1, RIP3, p-RIP1, p-RIP3, p62, and ROS. Necroptosis is usually detected with apoptosis, but other cell death modes are rarely detected.

**Table 5:** The necroptosis in PCa.

Active ingredients	Herbal	Real modules	Study type	Quality indicators	Dose	Pathway of action	Effects/mechanisms	Ref.
Curcumin	Curcuma	PCa models (cells+mice)	in vitro+in vivo	3 independent repeats (in vitro); 8–10 mice/group, 2 experiments (in vivo)	5–50 μM (in vitro), 50–200 mg/kg/day (in vivo)	PI3K/Akt/mTOR↓, NF-κB↓, AR↓, apoptosis regulators↑	Inducing necroptosis	(80)
20(S)-ginsenoside Rg3	Panax ginseng	Xenograft mouse model	in vivo	6–8 mice/group, 2 independent experiments	25–100 μM (likely mg/kg, keep as original)	RIPK1, RIPK3, MLKL↑, tumor weight↓	Inducing necroptosis	(81)
Shikonin (SHI)	Lithospermum erythrorhizon	PC-3, DU145, LNCaP, 22Rv1	in vitro	3 independent replicates, 3 wells each	0.1–1.5 μM	pRIP1↑, pRIP3↑	Inducing necroptosis	(82)

↑ = upregulated, ↓ = downregulated.

## The connection between PCD

PCD are essential for defending against diseases. Despite their distinct morphological and molecular characteristics, these pathways share commonalities. In PCa, autophagy and apoptosis exist in a dynamic balance of competition and cooperation. The effect of *thymus vulgaris* extract, DTX, CBD, XAP, QLD, bisbenzylisoquinoline alkaloids extracted, T-96, SA, Mistletoe, and PA on the autophagy and apoptosis are examined in the treatment of PCa. Autophagy typically serves as the first line of defense to repair damage and promote survival, but it fails to resolve cellular injury, apoptosis is activated to ensure controlled elimination. The interaction between Bcl-2 and beclin-1 establishes a crucial molecular link between apoptosis and autophagy. Beclin-1 is inherently susceptible to inhibition by Bcl-2, and the abundance of this interaction significantly influences the level of cellular autophagic activity (82). Disrupting the Bcl2/beclin-1 binding has emerged as an effective strategy to enhance autophagy (83, 84). Whereas controlled levels of autophagy promote cell survival and overactivation of autophagy accelerates apoptosis (85). The anti-autophagic and antiapoptotic effects of Bcl-2/Bcl-xL are regulated within the cell depending on their localization (86). Moreover, under certain cellular conditions, autophagy can promote cell death by either serving as an alternative cell death mechanism or enabling the induction of apoptosis. However, the interaction between the autophagy and apoptosis is not explored in the TPM for PCa.

**Figure 8:**
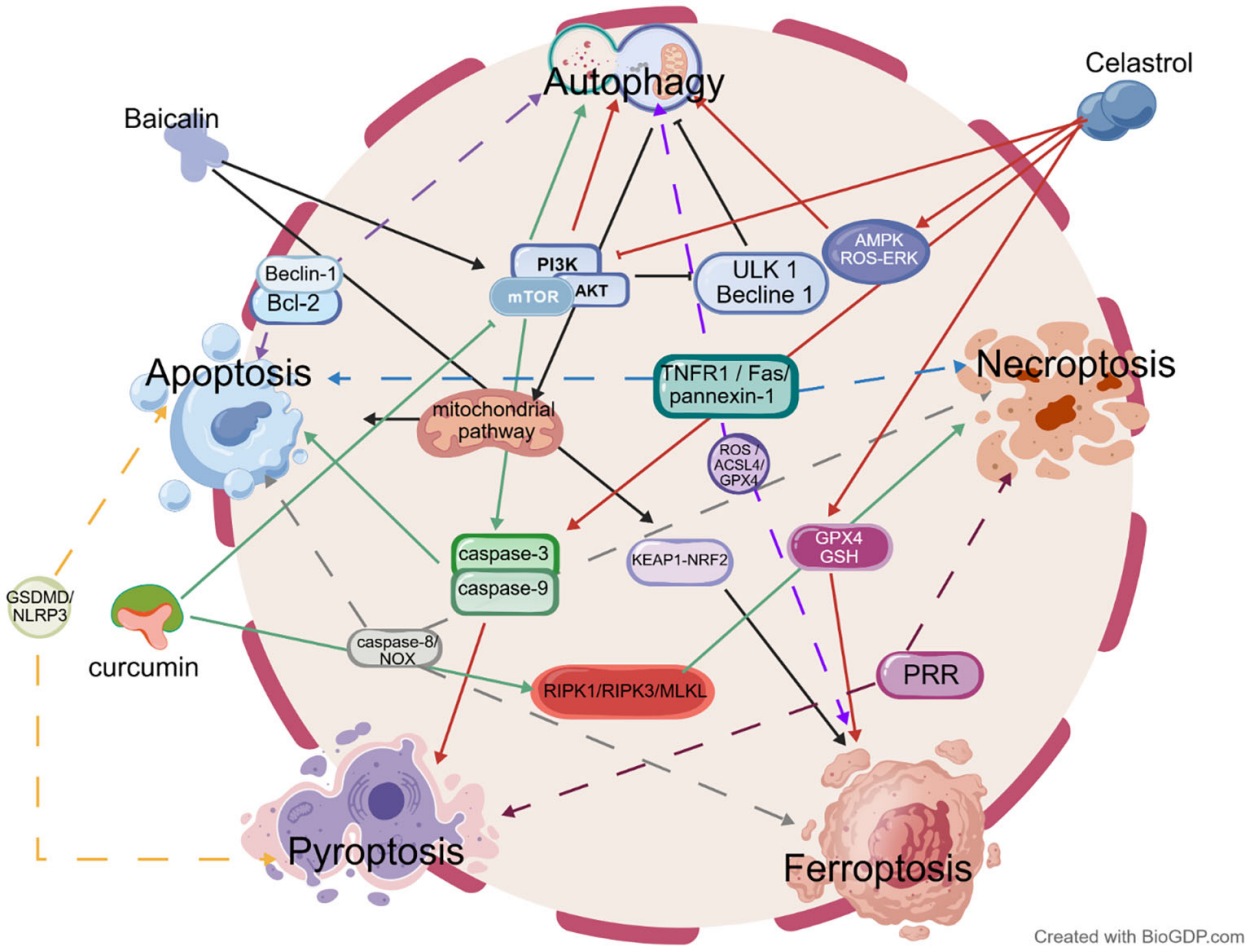
The connection between PCD in PCa. The effect of celastrol, curcumin, baicalin on the autophagy, proptosis, apoptosis and ferroptosis are detected in the treatment of PCa. Thus, celastrol, curcumin, and baicalin are used to reflect the connection between PCD. Baiclin increase PI3K/Akt, Keap1/Nrf2, promoting ferroptosis, autophagy and pyroptosis. Curcium inhibits PI3K/Akt, RIPK1/RIPK3/MLKL, promoting autophagy, apoptosis, and necroptosis. Celastrol increases AMPK/ROS/ERK, caspase 3, caspase 9, GPX4/GSH, and inhibits PI3K/Akt, promoting autophagy, pyroptosis, ferroptosis, apoptosis. Beclin-1/Bcl-2, GSDMD/NLRP3, PRR, Caspase 8/NOX regulate the relationship of autophagy/apoptosis, apoptosis/pyroptosis, pyroptosis/necroptosis, and apoptosis/necroptosis. Caspase 8/NOX regulates the relationship among the apoptosis, necroptosis and ferroptosis.

The apoptosis, necroptosis, and pyroptosis pathways have been shown to interact with each other. The effect of celastrol, curcumin, baicalin on the autophagy, proptosis, apoptosis and ferroptosis are detected in the treatment of PCa. The key targets are marked in the **Figure 8**. Pyroptosis shares PRRs-driven activation of signaling transduction with alternative necroptosis that responds to PAMPs and DAMPs (87). Pannexin-1, a channel-forming glycoprotein in macrophages, promoted NLRP3 inflammasome activation in the extrinsic and intrinsic apoptosis pathways (88). Necroptosis can also play an important role in initiating pyroptosis. Caspase families are extensively involved in apoptosis, necroptosis, and pyroptosis (44). Notably, the differential activation of caspases distinguishes the fate of cell death in various contexts. Caspase-1 is a crucial initiator of pyroptosis. However, it does not participate in apoptosis. In contrast, the downstream caspases involved in apoptosis, such as caspase-3 and caspase-7, inhibit pyroptosis by inactivating GSDMD. Caspase-8 can not only activate the downstream executioner caspase-3/7 in the apoptosis pathway but also cleave RIPK1 and RIPK3 in the ripoptosome of the necroptosis pathway, thereby preventing necroptosis and facilitating apoptosis (89). The effect of shikonin and butein on the necrosis and apoptosis are detected in the treatment of PCa. Extrinsic apoptosis and necroptosis are both initiated by the activation of cell surface death receptors, such as TNFR1 and Fas, which trigger intracellular signaling cascades involving adaptor proteins and kinases. The treatment of baicalin, curcuim and celastrol regulate three kinds of PCD, and the molecular connections between major PCDs in PCa were summarized to illustrate their intrinsic crosstalk.

Induction of ferroptosis may be a new treatment for PCa. The crosstalk between ferroptosis and apoptosis existed, and led to eventual cell death. The effect of artesunate, stachydrine hydrochlorid on the apoptosis and ferroptosis are detected in the treatment of PCa. As an important regulator of lipid redox signaling, NADPH oxidase (NOX) is shown to be involved in the stimulation of apoptosis (90). Similarly, NOX1-, NOX2-, and NOX4-mediated ROS production was also involved in initiating ferroptosis by inducing lipid peroxidation (88, 91, 92). The inactivation of acyl-CoA synthase long chain family member 4 (ACSL4) during apoptosis may inhibit the insertion of PUFAs into the cell membrane, thereby limiting the ability of cells to undergo ferroptosis. Autophagy is involved in the process of ferroptosis (93). The effect of GRh2 on the autophagy and ferroptosis are detected in the treatment of PCa. Oxidative stress and lipid peroxidation products are powerful inducers of autophagy, while excessive autophagy promotes ferroptosis (93, 94). Selective types of autophagy and chaperone-mediated GPX4 autophagy promoted ferroptosis by degrading ferroptosis repressors in a manner that increased intracellular free iron or enabled the accumulation of lipid peroxides (95).

## Conclusion and outlook

This review focuses on the anticancer mechanisms of TPM, clarifying the key targets of each cell death mode and providing references for subsequent research. The plant extracts, Chinese herbal compounds and pure compounds have been shown to induce apoptosis, ferroptosis, necroptosis PCa cell lines. However, the unclear mechanisms underlying the opposite autophagy and pyroptosis effects induced by different TPM. Few experiments have focused on PCD in the treatment of TPM in PCa. Paraptosis and cuproptosis should also attract more attention in the treatment of TPM for PCa. These cell death modes are often assessed alongside proliferation, migration, and invasion assays in PCa studies. Research has shown that TPM exerts anti-PCa effects through multi-component, multi-target, and multi-pathway characteristics, providing new ideas for clinical treatment. While preclinical efficacy is compelling, TPM’s translational trajectory remains impeded by pharmacokinetic constraints, and formulation standardization deficits. Hence, it is imperative to elucidate and explore the related molecular mechanisms of PCD in TPM therapeutic strategies for overcoming drug resistance in PCa or CRPC.
